# Supplementary material for: Association between toxic heavy metals and noncancerous thyroid disease: a scoping review
Source: PeerJ. 2025 Feb 11;13:e18962. doi: 10.7717/peerj.18962 (PMC11827576; doi:10.7717/peerj.18962)
Supplement: Supplemental Information 1 [file peerj-13-18962-s001.docx]

| No | Study | Study Location | Study Design | Biomarker heavy metal samples type |
| --- | --- | --- | --- | --- |
| 1 | Meeker et al 2009 | USA | Cohort | Blood |
| 2 | Christensen 2013 | USA | Cross-Sectional | Blood and Urine |
| 3 | Chen et al 2013 | USA | Cross-Sectional | Urine |
| 4 | Luo and Hendryx 2014 | USA | Cross-Sectional | Blood |
| 5 | Xu et al 2014 | China | Cross-Sectional | Blood |
| 6 | Akgol et al 2016 | Turkiye | Cross-sectional | Blood |
| 7 | Nie et al 2017 | China | Cross-sectional | Blood |
| 8 | Li et al 2017 | China | Cross-sectional | Blood |
| 9 | Yu et al 2017 | China | Animal experimental study | Blood |
| 10 | Luca et al 2017 | Italy | Animal experimental study | Blood |
| 11 | Nascimento et al 2018 | Brazil | Cross-sectional | Blood |
| 12 | Jurdziak 2018 | Poland | Cross-sectional | Urine |
| 13 | Afrifa et al 2018 | Ghana | Cross-sectional | Blood |
| 14 | Guo et al 2018 | China | Cross-sectional | Blood |
| 15 | Khan et al 2019 | Pakistan | Animal experimental study | Blood |
| 16 | Liao 2019 | USA | Cross-sectional | Urine |
| 17 | Maleki et al 2019 | Iran | Animal experimental study | Blood |
| 18 | Xu et al 2019 | China | Case-control study | Urine |
| 19 | Sun et al 2019 | China | Cross-sectional | Urine |
| 20 | Castiello et al 2020 | Spain | Cross-sectional | Urine |
| 21 | Wang et al 2020 | China | Cross-sectional | Urine |
| 22 | Fahim et al 2020 | Eqypt | Cross-sectional | Blood |
| 23 | De Lima Junior et al 2021 | Brazil | Animal experimental study | Blood |
| 24 | Kim et al 2021 | Korea | Cross-Sectional | Urine |
| 25 | Margetaki et al 2021 | Greece | Cross-Sectional | Urine |
| 26 | Campos et al 2021 | Brazil | Case-Control | Blood |
| 27 | Al Bazi et al 2021 | Saudi Arabia | Case-Control | Urine |
| 28 | Yalcin et al 2022 | Turkiye | Case-Control | Urine |
| 29 | Chen et al 2022 | China | Cross-Sectional | Blood |
