# Supplementary material for: Association between toxic heavy metals and noncancerous thyroid disease: a scoping review
Source: PeerJ. 2025 Feb 11;13:e18962. doi: 10.7717/peerj.18962 (PMC11827576; doi:10.7717/peerj.18962)
Supplement: Supplemental Information 2 — *Statistical analysis used. **Tested heavy metals but shows nonsignificant association with noncancerous thyroid illness in the study. [file peerj-13-18962-s002.docx]

| Study | Number of sample participants, n | Biomarker heavy metal samples (method of collection), analytical method | Findings |
| --- | --- | --- | --- |
| Meeker et al 2009 | Adult (n=219) | **Cr, As, Cd, Hg, Pb** samples (blood), inductively coupled mass spectrometry (ICP-MS) | ***Multiple linear regression models**  In the full Thyroid Stimulating Hormone (TSH) model, As was associated with an increase in TSH β =0.25 (0.03,0.47), and Pb was associated with decreases in TSH β = -0.19(-0.38, 0.004).  ****Nonsignificant heavy metals association: Cd, Cr, Hg** |
| Christensen 2013 | Adult  (n= 9762) | **Cd, Hg, Pb** samples (blood and urine), inductively coupled mass spectrometry (ICP-MS) | ***Multinomial regression analysis**  Cd measured in both blood adjusted β =−0.074 (0.028) and urine adjusted β = −0.119 (0.055) was associated with decreased TSH; Cd in urine was also associated with increased T3 adjusted β=0.033(0.008) and FT3 adjusted β = 0.012 (0.004). Similarly, Pb in both blood adjusted β = −0.028 (0.013) and urine adjusted β = −0.034 (0.013) was associated with decreased T4, and Hg in blood adjusted β =−0.013 (0.005) and urine adjusted β =−0.027 (0.012) associated with decreased total and free T3 and T4. |
| Chen et al 2013 | Adult (n=4409) | **Cd**, **Hg**, **Pb** samples (urine), inductively coupled mass spectrometry (ICP-MS) | ***Linear regression models**  Inverse association of T4 (β =–0.02 (–0.02, –0.01)) between Hg exposure and thyroid hormones, and a positive association T4 (β = 0.02 (0.001, 0.03)) between Cd exposure and thyroid hormones in adults. There was no association between thyroid hormones and Pb  ****Nonsignificant heavy metals association: Pb** |
| Luo and Hendryx 2014 | Adult  (n=7126) | **Cd, Pb** samples (blood), inductively coupled mass spectrometry (ICP-MS) | ***Multiple linear regression analysis**  The mean blood Cd was 0.55 μg/L (0.14–8.81) and the mean blood Pb was 1.82 μg/dL (0.18–33.10 μg/dL). Blood Cd was positively associated with free T4 only after adjustment for potential confounder. Blood Pb level was positively associated with free T3 only but no other thyroid hormones after adjustment for the same confounding variables |
| Xu et al 2014 | Children (n=167) | **Cd, Pb** samples (blood), inductively coupled mass spectrometry (ICP-MS) | ***Spearman rank correlation analysis and multiple stepwise regression analysis**  There was no correlation between the hormones and blood Pb or Cd levels found in the study  ****Nonsignificant heavy metals association: Cd, Pb** |
| Akgol et al 2016 | Adult (n=1724) | **Cd** samples (blood), inductively coupled mass spectrometry (ICP-MS) | ***Spearman correlation test**  There was a positive correlation between Cd and serum-free T4 and T3 levels (r=0.167, p< 0.001 and r=0.159, p<0.001, respectively). There was no correlation between whole blood Cd and serum TSH levels (r=0.026, p=0.826). |
| Nie et al 2017 | Adult (n=5628) | **Cd, Pb** samples (blood), atomic absorption spectrometer | ***Multivariate linear regression model**  The natural log(ln) Pb was positively related to the Thyroid Peroxidase Antibody (B ¼ 0.062, P < 0.05) and the Thyroid Stimulating Hormone) (B ¼ 0.047, P < 0.01) in women. The Cd in women was positively related to the Thyroglobulin Antibodies(TgAb) (B ¼ 0.046,  P < 0.05). In the adjusted logistic regression models, the Cd of women was positively related to their hypothyroid status and Thyroglobulin Antibodies (Tg Ab) tertiles. The ORs of women in the middle and higher Thyroid Peroxidase Antibody tertiles were 1.38 (P < 0.001) and 1.35 (P < 0.001) times greater for every unit increase in Pb, respectively. In men, no continuous correlation was found among variables. |
| Li et al 2017 | Adult  (n= 113) | **Pb** samples (blood) (inductively coupled mass spectrometry (ICP-MS) | ***Partial Correlation Analysis**  The level of Pb mean 1.84 μg/dL (95 % CI: 0.97, 2.70), was significantly lower in Nodular Goiter (p < 0.05). No significant correlations were observed between Pb and Free T3 or Free T4 in any group. |
| Yu et al 2017 | Rats (n= 7) | **Cd** samples (blood), atomic absorption spectrophotometer | ***Two-way ANOVA analysis**  CdCl2 does not influence the thyroid hormone levels significantly and no significant changes in thyroid coefficient were observed. There were no marked histopathological changes in the thyroid gland induced by Cd in treated samples. |
| Luca et al 2017 | Rats (n= 28) | **Cd** samples (blood), atomic absorption spectrophotometer | ***2-way unpaired t-test**  A significant increase in histological features of transformation was observed in thyroid follicular cells of rats treated with Cd compared with those of the control group. Slightly increased environmental concentrations of Cd can accelerate the appearance of transformation marks in the thyroid gland of hypothyroid rats. |
| Nascimento et al 2018 | Children (n=54) | **Cr, As, Cd, Hg, Pb** samples (blood) (inductively coupled mass spectrometry (ICP-MS) | ***Spearman’s rank correlation analysis and partial correlation analysis with a log link**  Blood levels of chromium (Cr) (r= 0.476, p<0.01) and lead (Pb) (r= 0.376, p<0.05) were positively correlated with thyroid stimulating hormone (TSH) concentrations and negatively associated with free thyroxine (fT4) levels in the low exposure period  ****Nonsignificant heavy metals association: As, Cd, Hg** |
| Jurdziak 2018 | Smelting workers (n=102) | **Cd, Pb** (blood), **As** samples (urine), inductively coupled mass spectrometry (ICP-MS) | ***Logistic Regression Analysis**  Logistic regression demonstrated that higher Cd (aOR = 1.532; p = 0.027) represented independent risk factors of abnormal values of TSH in this group.  ****Nonsignificant heavy metals association: Pb, As** |
| Afrifa et al 2018 | Male gold miner (n=137) | **Hg** (blood), cold vapor atomic absorption spectrophotometer (CVAAS) | ***Spearman rho moment correlation analysis**  Blood Hg showed negative correlation with T3(r = -0.29, P<0.0001), and T4(r =-0.69, P<0.0001) and positive correlation with work duration (r = 0.88, P<0.001). Even though a positive trend of association between blood Hg and TSH levels was recorded, it was not significant (r = 0.07, P = 0.4121) |
| Guo et al 2018 | Pregnant women (n=915) | **Cr, As, Cd, Hg, Pb** samples (blood), inductively coupled mass spectrometry (ICP-MS) | ***Multiple linear regression analysis**  Only Arsenic (As) was significantly linked to decreased levels of one or more thyroid hormones (T3= −0.95% (−3.70%, 1.88%), FT3 = −0.11% (−2.14%, 1.96%)) based on trend tests in the single-metal models.  ****Nonsignificant heavy metals association: Cr, Cd, Hg, Pb** |
| Khan et al 2019 | Rabbit  (n= 66) | **Cd**, **Hg** samples (blood), atomic absorption spectrophotometer | ***One-way ANOVA, with “Dunnett’s” Multiple Comparison Test**  **3,3,5-triiodothyronine (T3) concentration**  The level of serum T3 in the Cd exposed group (0.4 ± 0.0 ng/ml) showed the highest significant decrease while in the Hg exposed (0.4 ± 0.0 ng/ml) and combination of Cd + Hg group animals (0.3 ± 0.0 ng/ml), it showed a higher and the highest significant difference.  **Thyroxine (T4) concentration**  The level of serum T4 in Cd (26.3 ± 1.6 ng/ml), Hg (21.3 ± 1.1 ng/ml), and co-administration of Cd + Hg (27.3 ± 1.3 ng/ml) showed a comparable significant decrease  **Thyroid-stimulating hormone concentration (TSH) concentration**  Level of serum TSH in Cd (0.17 ± 0.01 nmol/l), Hg (0.19 ± 0.01 nmol/l), and coadministration of Cd + Hg (0.17 ± 0.01 nmol/l) showed the highest significant decrease difference |
| Liao 2019 | Adult (n= 4207) | **As, Cd, Pb** (urine), inductively coupled mass spectrometry (ICP-MS) | ***Multiple linear regression analysis**  The study found that urinary Cd (OR: 2.05, 95% CI: 1.03, 4.06) was significantly associated with increased odds of thyroid dysfunctions (hypothyroidism and hyperthyroidism). There was no significant association between Pb, with thyroid dysfunctions.  ****Nonsignificant heavy metals association: As, Pb** |
| Maleki et al 2019 | Rats (n= 45) | **As, Cd, Hg, Pb** samples (blood), atomic absorption spectrophotometer | ***One-way ANOVA analysis**  The number of heavy metals like Pb, Cd, As, and Hg showed a significant increase in the serum of animals in the treatment group compared to negative control and control groups (P ≤ 0.05). Serum levels of thyroid and parathyroid hormone in the treatment group showed a significant decrease compared to control groups (P ≤ 0.05). In addition, histological investigations demonstrated relative changes in tissue and functional structures of important thyroid and parathyroid glands tissues. |
| Xu et al 2019 | Children (n=176) | **Cr, Cd, Pb** samples (urine), inductively coupled mass spectrometry (ICP-MS) | ***Partial correlation analysis**  The mean blood levels of Cr, Cd, and Pb in the exposure group were all statistically higher than in the control group (2.57 vs. 0.79 mg/L; 1.83 vs. 1.81 mg/L; 44.00 vs. 32.31 mg/L,p < 0.01) Moreover, mean serum levels of FT3, FT4, T3, T4, and TSH between the two groups all demonstrated no significant differences (P > 0.05).  ****Nonsignificant heavy metals association: Cr, Cd, Pb** |
| Sun et al 2019 | Pregnant women (n=675) | **Cr, As, Cd, Pb** samples (urine), inductively coupled mass spectrometry (ICP-MS) | ***Multivariable linear regressions analysis**  Only urinary As and Pb concentrations exhibited inverse associations with the FT3 or FT3/FT4 ratio. For each unit increase in ln-transformed urinary As and Pb levels, there was a 0.015 and 0.011 lowering in serum ln-FT3. There were no observed relationships between any urinary metal and serum TSH and FT4 concentrations.  ****Nonsignificant heavy metals association: Cr, Cd** |
| Castiello et al 2020 | Adolescents (n= 155) | **Cr, As, Cd, Hg, Pb** samples (urine), inductively coupled mass spectrometry (ICP-MS) | ***Multivariate linear regression analysis**  There were significant associations of Cr with decreased TSH (-24, 95%CI=-42; -1). A significant inverse association between Hg and TSH (-4; 95%CI=-8; -1 for each 50% increase in Hg) was also observed. There was no significant association between Cd, Pb, and Ar with thyroid hormones.  ****Nonsignificant heavy metals association: As, Cd, Pb** |
| Wang et al 2020 | Pregnant women (n=910) | **As, Cd, Hg, s**amples (urine), inductively coupled mass spectrometry (ICP-MS) | ***Multiple adjusted linear regression models**  Hg (> 0.162 μg/L), Cd (> 0.084 μg/L), and As (> 0.348 μg/L) were detectable in 76.9%, 90.1%, and 100% of maternal urine samples from women in the first trimester of pregnancy. Maternal exposures to Cd in the first trimester were positively associated with neonatal TSH levels (P = 0.04). There were no significant associations between Hg and As with neonatal TSH levels.  ****Nonsignificant heavy metals association: As, Hg** |
| Fahim et al 2020 | Foundry worker (n= 87) | **Pb** samples (blood) inductively coupled plasma emission spectrometer (ICP-OES) | ***Pearson correlation coefficient**  Mean blood Pb levels (16.5±1.74 μg/dl) were significantly higher among the exposed workers compared to those of the control group (12.8±1.16 μg/dl, (p <0.001)). The exposed group had significantly increased free triiodothyronine (FT3), free thyroxine (FT4) (p<0.0001) and significantly decreased thyroid stimulating hormone (TSH) with mean (1.77±0.44 μIU/ml) (p<0.0001). |
| De Lima Junior et al 2021 | Rats (n= 60) | **Pb** samples (blood), inductively coupled mass spectrometry (ICP-MS) | ***One-way ANOVA analysis**  Serum levels of T4 increased 25.7% (p < 0.05) in the 25 mg/kg male group and in both female groups treated with 10 mg/kg and 25 mg/kg of lead acetate (58.8% e 52%, p <0.05) T3 serum levels were reduced 50.9% in the male group treated with 10 mg/kg of lead acetate and 20.3% in the female group treated with 25 mg/kg when compared to the respective control groups TSH serum levels did not suffer statistically significant changes Serum levels of lead in controls and all lead-treated groups were under the detection threshold of the technique |
| Kim et al 2021 | Adult (n=3787) | **Cd, Hg, Pb** samples (urine), inductively coupled mass spectrometry (ICP-MS) | ***Multiple linear regression analysis**  Urinary Hg was negatively associated with total T3 (β=−0.032, p<0.001), while it was positively associated with total T4 among females only (β = 0.031, p = 0.016). In contrast, urinary Cd was positively associated with total T3 in both male and female populations (β = 0.033, p < 0.001 in males, and β = 0.032, p < 0.001 in females). Urinary Cd also showed a positive association with thyroid autoantibodies, but only in males. There was no observation between Pb and thyroid hormones  ****Nonsignificant heavy metals association: Pb** |
| Margetaki et al 2021 | Pregnant Women (n=824) | **Cd**, **Pb** samples (urine), inductively coupled mass spectrometry (ICP-MS) | ***Bayesian Kernel Machine Regression (BKMR)**  Women with high (3rd tertile) concentrations of urinary Cd had 13.3 % (95%CI: 2.0 %, 23.2 %) lower TSH compared to women with low concentrations (2nd and 1st tertile). In addition, women with high urinary Cd had 2.2 % (95%CI: 0.0 %, 4.4 %) higher fT4 and 4.0 % (95%CI: 0.1 %, 8.1 %) higher fT3 levels, and women with high urinary Pb had 4 % (95%CI: 0.2 %, 8.0 %) higher fT3 levels compared to women with low exposure. |
| Campos et al 2021 | Adult (n=1550) | **As, Cd, Hg, Pb,** samples (blood), inductively coupled mass spectrometry (ICP-MS) | ***One-way ANOVA or Mann-Whitney test**  Toxic metals geometric mean concentrations in whole blood and plasma were 4.82 and 8.33g/L for As; 0.25 and 0.03 g/L for Cd; 0.92 and 0.17 g/L for Hg; and 20.8 and 1.46 g/L for Pb, respectively. There was no association between the heavy metals examined with the thyroid hormones.  ****Nonsignificant heavy metals association: As, Cd, Hg, Pb.** |
| Al Bazi et al 2021 | Adult  (n=100) | **Cr,** **Cd,** (urine), inductively coupled mass spectrometry (ICP-MS) | ***Pearson’s correlation**  Urine from patients with thyroid disorders (hyperthyroidism and hypothyroidism) had significantly higher concentrations of Cd only with a mean (0.024± 0.001 p-values <0.0005). There were also non-significant correlations between T3 and T4 levels and all trace elements  ****Nonsignificant heavy metals association: Cr** |
| Yalcin et al 2022 | Adolescents (n= 42) | **As, Cd**, **Hg**, **Pb**, samples (urine), inductively coupled mass spectrometry (ICP-MS) | ***Logistic Regression Analysis**  Adolescents having the highest tertile of Hg had 21.3 times more Thyroid Cysts than the first tertile (CI: 2.2–207.0). The risk for Thyroid Cysts did not change within the tertile groups of Cd, Pb, and Ar.  ****Nonsignificant heavy metals association: As, Cd, Pb** |
| Chen et al 2022 | Adult  (n= 2877) | **Cd, Pb** samples (blood), graphite furnace atomic absorption spectrometry  (GFAAS) | ***Logistic regression models**  The fourth quartiles of Pb were positively associated with thyroid peroxidase antibody (OR 1.637, p=0.006), Antithyroid antibody (OR 1.435, p=0.025), hypothyroid status (OR 1.467, p=0.013) and TSH levels (B 0.092, p=0.021). The fourth quartile of Cd was positively associated with the prevalence of antithyroid antibody (OR 1.427, p=0.036). |
