# Supplementary material for: Association between toxic heavy metals and noncancerous thyroid disease: a scoping review
Source: PeerJ. 2025 Feb 11;13:e18962. doi: 10.7717/peerj.18962 (PMC11827576; doi:10.7717/peerj.18962)
Supplement: Supplemental Information 4 [file peerj-13-18962-s004.docx]

**Identification of studies via databases and registers**

Records removed *before screening*:

Duplicate records removed (n = 216)

Records marked as ineligible by automation tools (n =136)

Records identified from*:

Databases (n = 552)

PubMed (n= 130)

Web Of Science (n= 197)

Scopus (n=225)

**Identification**

Records excluded**

(n = 300)

Different population (animal) (n= 32)

Irrelevant title (n= 132)

Not thyroid disease-related (n= 42)

Thyroid cancer (n= 21)

Review (n=61)

Other heavy metal types (n= 12)

Records screened

(n = 336)

Reports sought for retrieval

(n = 36)

**Screening**

Reports assessed for eligibility

(n = 36)

Reports excluded: (n=7)

Conference (n = 2)

Non-English (n = 3)

Proceeding (n = 2)

Studies included in review

(n = 29)

Reports of included studies

(n = 29)

**Included**

*Consider, if feasible to do so, reporting the number of records identified from each database or register searched (rather than the total number across all databases/registers).

**If automation tools were used, indicate how many records were excluded by a human and how many were excluded by automation tools.

*From:*  Page MJ, McKenzie JE, Bossuyt PM, Boutron I, Hoffmann TC, Mulrow CD, et al. The PRISMA 2020 statement: an updated guideline for reporting systematic reviews. BMJ 2021;372:n71. doi: 10.1136/bmj.n71. For more information, visit: <http://www.prisma-statement.org/>
